# Development of a rapid and novel diagnostic technique for cardiac amyloidosis using Raman spectroscopy

**DOI:** 10.21203/rs.3.rs-6795517/v1

**Published:** 2025-06-25

**Authors:** Mizuki Yoshimoto, Shin-ichiro Yanagiya, Hiroki Takanari, Takeshi Honda, Yusaku Maeda, Ryohei Sumitani, Masahiro Oura, Kimiko Sogabe, Takeshi Harada, Shiro Fujii, Shingen Nakamura, Yoshimi Bando, Koichi Tsuneyama, Itsuro Endo, Masahiro Abe, Ken-ichi Matsuoka, Hirokazu Miki

**Affiliations:** Faculty of Science and Technology, Tokushima University; Department of Next Generation Photonics, Institute of Post-LED Photonics; Department of Legal Medicine, Nihon University School of Medicine; Graduate School of Technology, Industrial and Social Sciences, Tokushima University; Department of Hematology, Endocrinology and Metabolism, Tokushima University Graduate School of Biomedical Sciences; Department of Hematology, Endocrinology and Metabolism, Tokushima University Graduate School of Biomedical Sciences; Department of Hematology, Endocrinology and Metabolism, Tokushima University Graduate School of Biomedical Sciences; Department of Hematology, Endocrinology and Metabolism, Tokushima University Graduate School of Biomedical Sciences; Department of Hematology, Endocrinology and Metabolism, Tokushima University Graduate School of Biomedical Sciences; Department of Hematology, Endocrinology and Metabolism, Tokushima University Graduate School of Biomedical Sciences; Department of Community Medicine and Medical Science, Tokushima University Graduate School of Biomedical Sciences; Division of Pathology, Tokushima University Hospital; Department of Pathology and Laboratory Medicine, Tokushima University Graduate School of Biomedical Sciences; Department of Bioregulatory Sciences, Tokushima University Graduate School of Biomedical Sciences; Department of Hematology, Kawashima Hospital; Department of Hematology, Endocrinology and Metabolism, Tokushima University Graduate School of Biomedical Sciences; Division of Transfusion Medicine and Cell Therapy, Tokushima University Hospital

**Keywords:** Cardiac amyloidosis, Rapid diagnosis, Immunoglobulin light chains, Transthyretin, Raman spectroscopy

## Abstract

Although the prognosis of cardiac amyloidosis has improved with the development of therapies, the time required for disease typing remains a critical issue. We investigated the potential of Raman spectroscopy for the more rapid diagnosis and typing of cardiac amyloidosis. Heart biopsies were collected from patients with the AL (4) and ATTR (4) types of cardiac amyloidosis, and tissue sections were subjected to Raman microscopy. A principal component analysis (PCA) of spectral data was performed and receiver operating characteristic (ROC) curves were created to confirm the accuracy of discriminating between amyloid-deposition and non-deposition sites, and between AL and ATTR. The steep peak at 1680 cm^−1^, reflecting the β-sheet structure, was useful for detecting the amyloid-deposition region. By restricting the spectral analysis to amyloid-deposition sites, AL and ATTR were discriminated by principal components with a characteristic broad peak at 1520–1540 cm^−1^, which was also observed in the Raman spectrum of AL, but not ATTR. The area under ROC curve discriminating AL and ATTR was 0.78. PCA of the Raman spectra of cardiac biopsies has the potential not only to detect amyloid-deposition sites in tissue but also to rapidly discriminate between the AL and ATTR types of cardiac amyloidosis.

## Introduction

Amyloidosis is the general term used for diseases in which amyloid proteins with an abnormal three-dimensional structure are deposited in tissues, causing organ damage and dysfunction. There are more than 40 known precursor proteins that produce amyloid proteins, and the types of amyloid proteins deposited in organs differ and cause specific symptoms. The pathophysiological condition of amyloid protein accumulation in the heart is called cardiac amyloidosis. Typical precursor proteins that give rise to cardiac amyloidosis are immunoglobulin light chains and transthyretin, termed the AL and ATTR types, respectively. Cardiac amyloidosis has a poor prognosis with many cases of sudden death due to pump failure and fatal arrhythmias, especially in AL, where the frequency of fatal complications is reported to be high^1^. In recent years, several new agents have been developed to improve the prognosis of the AL and ATTR types of cardiac amyloidosis. Daratumumab is a human IgG-κ monoclonal antibody that targets CD38, which is highly expressed on human plasma cells. Recent clinical studies showed that daratumumab combined with chemotherapy achieved better event-free survival and hematological responses in newly diagnosed systemic AL amyloidosis patients, including cardiac amyloidosis^2–4^. Tafamidis, developed as a pharmacological chaperone, improved the prognosis of patients with ATTR cardiac amyloidosis by stabilizing the tetrameric structure of transthyretin and inhibiting its tissue deposition^5,6^. In current clinical practice, when amyloidosis is suspected, several biopsy samples are taken from patients, and if amyloid deposition is detected, the disease type is identified via immunostaining and a genetic diagnosis, which is very time-consuming, taking several weeks to several months. In this context where effective treatments are being developed, a more rapid diagnosis to identify the type of cardiac amyloidosis is needed.

Raman spectroscopy is a label-free optical measurement technique that assesses molecular structures by measuring the spectrum of scattered light induced by single-wavelength laser irradiation and detecting the wavenumber shift from irradiated light^7^. Each molecule has a unique “spectral fingerprint” depending on vibrating patterns, which may be identified by chemical bonds and three-dimensional structures. Raman spectroscopy has recently been used in analyses in biological and medical research^8–10^. It is a non-destructive technique that does not depend on the state of samples, which enables its application to live organs. Raman spectroscopy is useful for detecting tumors^11–13^, myocardial infarction^14^, skin inflammation^15^ and amyloid-β protein in Alzheimer’s disease^16–18^. Raman spectroscopy is also increasingly used as a surgery-assisting tool^19,20^. In recent years, many attempts have been made to improve diagnostic accuracy by using artificial intelligence (AI) or machine learning to analyze spectra obtained by Raman spectroscopy^15,21,22^. However, living organs comprise a wide variety of molecules, each of which produces scattered light with multiple Raman shifts, and when these overlap, it becomes difficult to identify single molecules. On the other hand, in pathologies such as amyloidosis, a single substance aggregates densely in tissue and, thus, it is easier to detect. We previously examined clinical biopsy specimens from patients with amyloidosis and reported that optical mapping based on Raman spectra derived from the β-sheet structure, which is abundant in amyloid protein, detected amyloid-deposition sites^23^. Our findings demonstrated the potential of a Raman spectroscopic analysis to more rapidly diagnose amyloid deposition in tissues. However, it remains unclear whether it has the ability to detect differences in disease types. Therefore, with the aim of rapidly diagnosing the disease type of cardiac amyloidosis, we herein analyzed spectra in more detail to establish whether a Raman spectral analysis discriminates between the AL and ATTR types of cardiac amyloidosis.

## Results

### Patient background

Table 1 shows the summarized data of patients enrolled in the present study. Between 2018 and 2024 we treated 4 AL and 4 ATTR cardiac amyloidosis patients. Although the small number of patients who participated during the current study period makes it difficult to examine the significance of differences, the followings were observed. 1) Patients with ATTR amyloidosis were slightly older than those with AL amyloidosis. 2) The NYHA functional classification, which represents the clinical severity of heart failure, was higher in AL amyloidosis than in ATTR amyloidosis, indicating that AL amyloidosis resulted in more severe heart failure. 3) The thicknesses of interventricular septum and left ventricular posterior wall in echocardiography, which indicate myocardial hypertrophy, were slightly wider in ATTR, suggesting that cardiac hypertrophy was more intense in ATTR cardiac amyloidosis. 4) The serum level of brain natriuretic peptide, a biomarker of heart failure, was slightly higher in AL amyloidosis, also suggesting that heart failure was more severe in the AL type of cardiac amyloidosis than in the ATTR type. Such patient background was well consistent with previous reports of sudden death in AL and ATTR types of cardiac amyloidosis^1^.

### Raman imaging and Raman spectra of AL and ATTR cardiac amyloidosis

**Figure 1:**
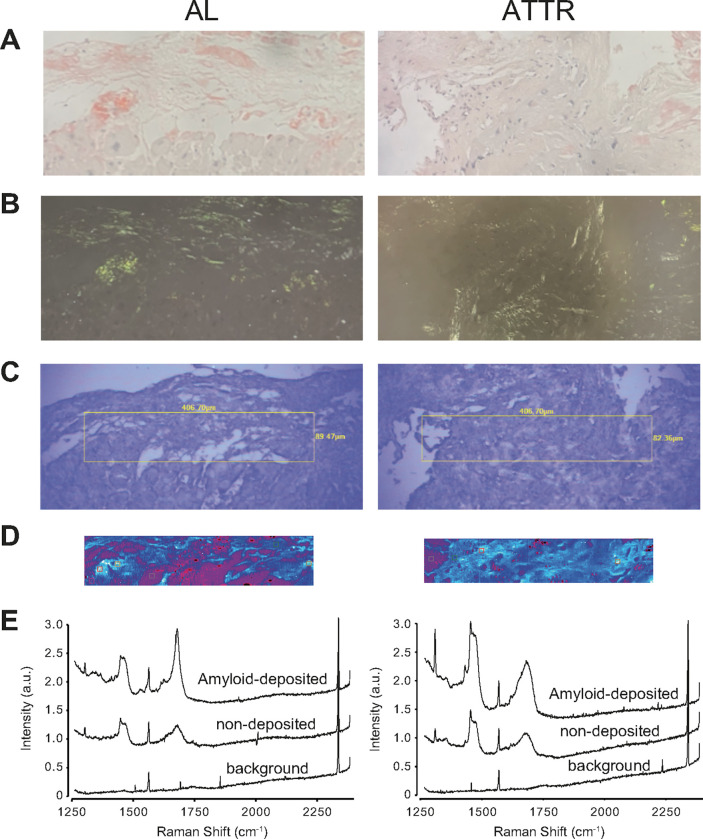
Representative histological findings and spectral data of AL (left) and ATTR (right) cardiac biopsy samples. ***A:*** Transmitted light microscopy images of Congo Red stained specimens. ***B:*** Polarized light microscopy images of Congo Red stained specimens. ***C:*** Transmitted light microscopy images of adjacent unstained specimens. Yellow boxes indicate the area where Raman spectra were acquired and Raman images were created. ***D:*** Raman images created from Raman spectra obtained from the yellow-framed region of unstained specimens in panel *C*. The ratio of the Raman scattering intensities in the wavenumber range of 1665–1680 cm^−1^ to that in the wavenumber range of 1580–1600 cm^−1^ was calculated, and the image was displayed with a pseudo-color gradient of black, magenta, cyan, and white from the lowest to highest ratio. ***E:*** Raman spectra recorded in the white (amyloid-deposition area), cyan (non-deposition area), and magenta (background) pixels of the Raman image in panel *D* are shown from the top to bottom. AL, amyloid light-chain; ATTR, amyloidosis of transthyretin

Representative transmitted light microscopy images (Fig. 1A) and polarized light microscopy images (Fig. 1B) of Congo-Red-stained specimens as well as transmitted light microscopic images of unstained specimens (Fig. 1C), Raman images (Fig. 1D) and Raman spectra (Fig. 1E) obtained from adjacent unstained specimens are shown. Similar to our previous findings^23^, amyloid-deposition areas that stain red with Congo-Red and exhibited apple green birefringence in polarized light microscopy were shown in white in the Raman image, indicating that Raman intensity at 1680 cm^− 1^ was very strong. Additionally, the peak at 1680 cm^− 1^, which was assigned to an amide I bond, was clearly visible at the amyloid deposition sites of AL and ATTR cardiac amyloidosis. This was consistent with our previous findings^23^.

### Detection of amyloid-deposition regions in heart tissue based on principal component analysis (PCA) of Raman spectra

**Figure 2:**
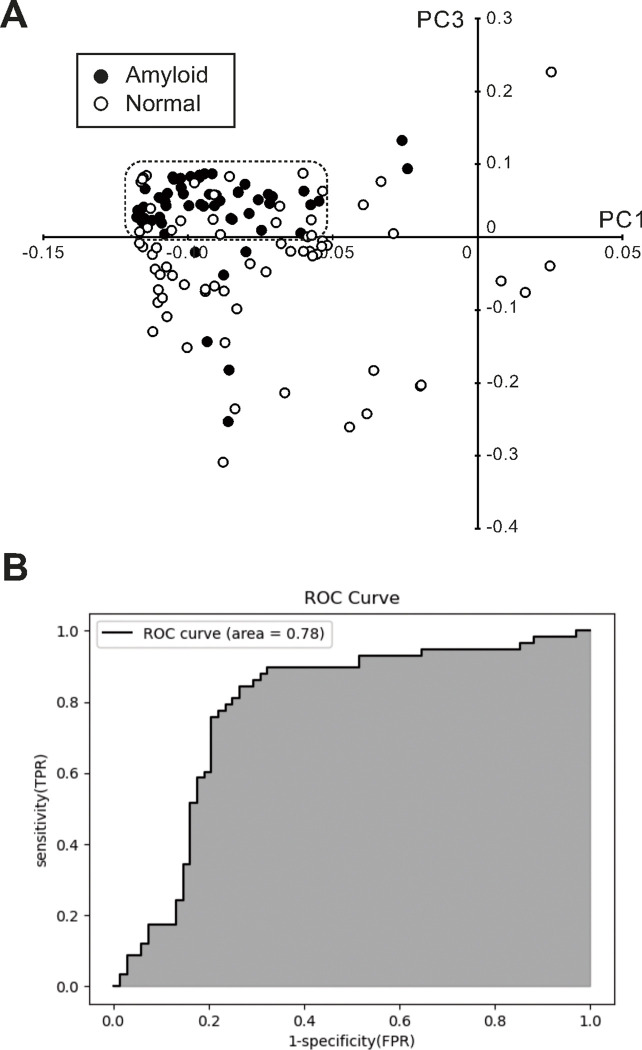
Discrimination between amyloid-deposition and non-deposition regions based on PCA. ***A:*** Scatter plots created by PC1 and PC3 obtained by PCA of the Raman spectra of amyloid-deposition (closed circles) and non-deposition (open circles) regions. The dotted box indicates the area where data from amyloiddeposition regions predominantly accumulated. ***B:*** ROC curve for the detection of amyloid-deposition regions by PC1 and PC3 obtained by PCA of the Raman spectra of amyloid-deposition and non-deposition regions. PCA, principal component analysis; PC principal component; ROC curve, receiver operating characteristic curve

To assess differences more objectively in Raman spectra between normal and amyloid-deposition areas, we performed PCA of total Raman spectral data to create scatter plots of two different principal components (PCs). Figure 2A shows a scatterplot based on PC1 and PC3. Although there was some overlap, plots of the amyloid deposition areas (closed dots) were more likely to be clustered in the dotted box, while those of normal tissue areas (open dots) were more likely to be dispersed around them. To confirm whether the results of PCA of Raman spectra discriminated between amyloid-deposition and non-deposition regions, we created a receiver operating characteristic (ROC) curve based on PC3 obtained by PCA. The area under the curve (AUC), sensitivity, and specificity were 0.78, 0.84, and 0.74, respectively.

We also tested the ability to identify amyloid deposition sites based on Raman spectra separately for AL and ATTR cardiac amyloidosis. Figure S1A is a scatter plot based on the results of PCA of Raman spectroscopy of AL cardiac amyloidosis. Although there was some overlap, the distribution of the plots of amyloid deposition area (orange dots) and normal area (open dots) appeared to differ. The ROC curve based on PCA results showed AUC of 0.69, sensitivity of 0.87, and specificity of 0.59 (Fig. S1A). Figure S2A is a scatterplot based on the results of PCA of Raman spectroscopy of ATTR cardiac amyloidosis. Although there was some overlap, the distribution of plots of the amyloid deposition area (green dots) and normal area (open dots) appeared to differ. The ROC curve based on PCA results showed that AUC, sensitivity, and specificity were 0.77, 0.75, and 0.88, respectively (Fig. S2B). These results suggest the potential to detect amyloid-deposition sites with high accuracy based on the PCA of Raman spectroscopy.

### Discrimination of AL and ATTR cardiac amyloidosis based on principal component analysis of Raman spectra.

**Figure 3:**
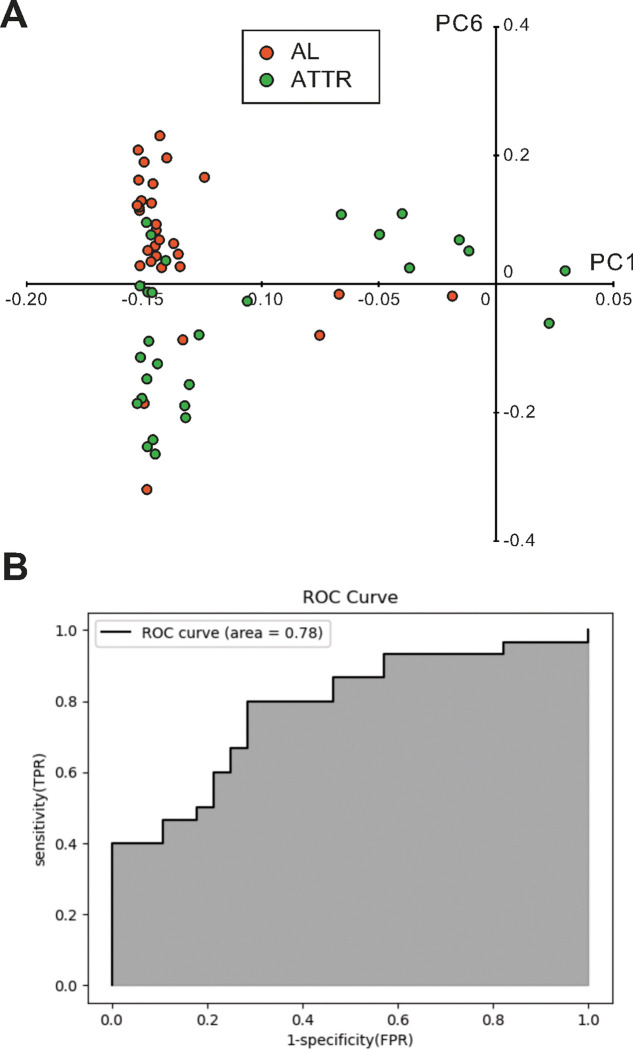
Discrimination between AL and ATTR cardiac amyloidosis based on PCA. ***A:*** Scatter plots created by PC1 and PC6 obtained by performing PCA of the Raman spectra of amyloid-deposition regions in AL (orange circles) and ATTR (green circles) specimens. ***B:*** ROC curve for the detection of AL by PC6 obtained by PCA of the Raman spectra of AL and ATTR samples. AL, amyloid light-chain; ATTR, amyloidosis of transthyretin; PCA, principal component analysis; PC principal component; ROC curve, receiver operating characteristic curve

We performed PCA using only data obtained from amyloid deposition regions in both AL and ATTR cardiac amyloidosis to investigate whether it is possible to distinguish between the AL and ATTR types. Figure 3A shows a scatterplot of PC1 and PC6 based on the PCA of data limited to amyloid deposition areas. Although there was a slight overlap, AL (orange dots) and ATTR (green dots) were separated by positive and negative values of PC6, respectively. Figure 3B shows the ROC curve generated to confirm whether AL and ATTR may be discriminated based on the results of the PCA of Raman spectra obtained at amyloidosis deposition areas. AUC, sensitivity, and specificity were 0.78, 0.80, and 0.71, respectively.

**Figure 4:**
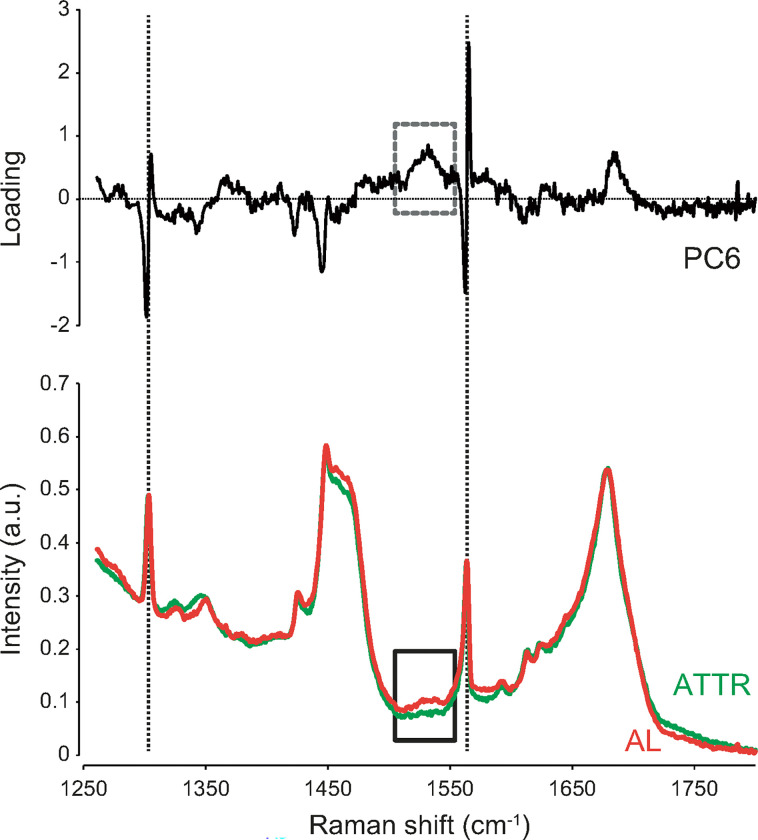
Detailed comparison of Raman spectra obtained from AL and ATTR cardiac amyloidosis samples. The averaged waveforms of Raman spectra from AL (orange line) and ATTR (green line) cardiac amyloidosis samples were compared side-by-side with the loading waveform of PC6 obtained by PCA of the Raman spectra of amyloid-deposition regions in AL and ATTR. The solid box shows an apparent difference in Raman intensity between AL and ATTR. An upward broad wave was observed in the loading waveform of PC6 in the same wavenumber region (dotted box). AL, amyloid light-chain; ATTR, amyloidosis of transthyretin; PC principal component; PCA, principal component analysis

In Fig. 4, the average spectral waveforms of the amyloid deposition regions in AL and ATTR cardiac amyloidosis were compared side by side with the loading waveform of PC6 in the wavenumber range of 1250–1800 cm^− 1^. A difference was observed between the Raman spectral intensities of AL and ATTR in the range of 1520–1540 cm^− 1^ (solid box). Multiple peak-fitting in the range of 1500–1550 cm^− 1^ revealed a typical peak at 1537 cm^− 1^ in the spectrum of AL, but not ATTR. The loading wave of PC6 obtained by the PCA of Raman spectra in amyloid-deposition regions shows an upward broad peak at 1537 cm^− 1^ (dotted box). The spectral difference in 1520–1540 cm^− 1^ was considered to contribute to the highly accurate discrimination of AL and ATTR based on PC6.

**Figure 5:**
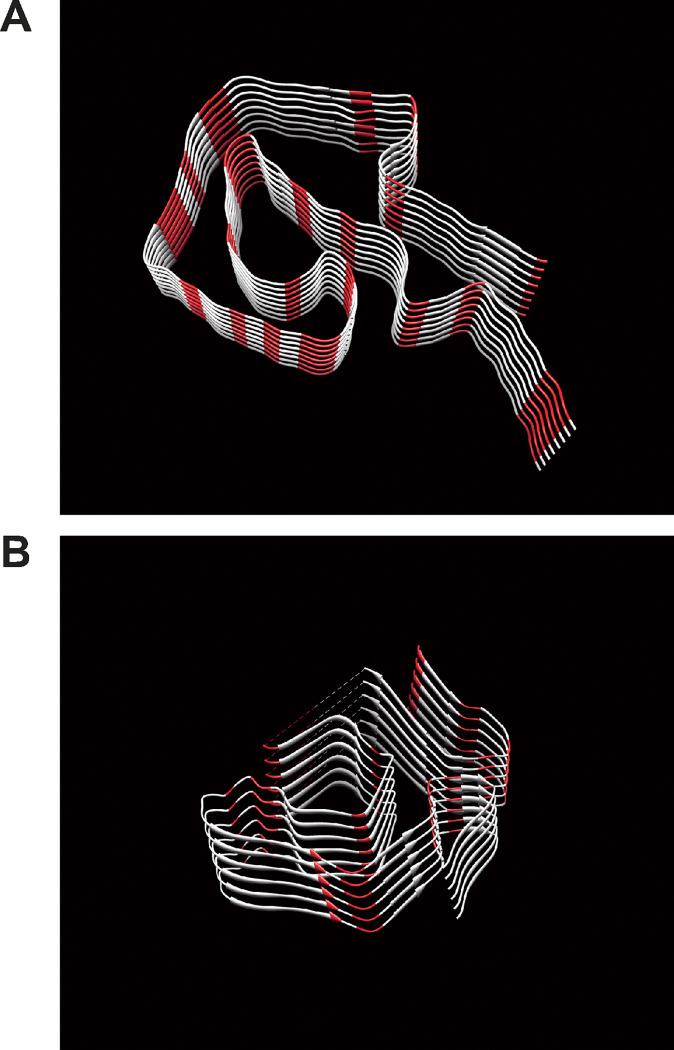
Three-dimensional structures of amyloid proteins comprising immunoglobulin (A) and transthyretin (B). The amino acid sequence in the amyloid-forming region of each protein is based on previous studies^24,25^. Amino acids with Raman spectra around 1537 cm^−1^ are colored in red^26,27^.

We then investigated whether the difference in the Raman peak at 1537 cm^− 1^ in amyloid-deposition regions in AL and ATTR was due to variations in the amino acid content of their precursor proteins. Figure 5 shows the three-dimensional structure of amyloid fibrils consisting of immunoglobulin light chains (lambda type) with the IGLV1–44 mutation, which is one of the most common mutations observed in AL amyloidosis^24^, and wild-type transthyretin^25^. The amino acid sequences of both precursor proteins are shown in Figure S3, and the content of amino acids in both proteins are also summarized in Table S1 in supplementary data. According to previous studies^26,27^, among the 20 amino acids that make up the human body, those with peaks around the wavenumber of 1538 cm^− 1^ were glycine (1515 cm^− 1^), asparagine (1541 cm^− 1^), isoleucine (1547 cm^− 1^), leucine (1514 cm^− 1^), proline (1548 cm^− 1^), and tryptophan (1559 cm^− 1^). The total numbers of the six amino acids in the amyloid-forming portion of immunoglobulin light chains and transthyretin were 31 (34%) and 29 (26%), respectively. Their content differed by 8%. As shown in Fig. 5, the content of six amino acids colored red differed and also appeared to be denser and in closer proximity in immunoglobulin light chains than in transthyretin. The different amino acid sequences of the precursor proteins may be responsible for the differences observed in Raman spectra in amyloid-deposition areas of the two disease types of cardiac amyloidosis.

## Discussion

We successfully performed Raman spectroscopy on formalin-fixed paraffin-embedded sections of myocardial biopsy samples from patients with cardiac amyloidosis to detect sites of amyloid deposition in cardiac tissue. Furthermore, a detailed Raman spectral analysis using PCA revealed the potential to distinguish between the two most common forms of amyloid deposition in the heart, AL and ATTR.

Since Raman spectroscopy is an optical analysis that is non-destructive and does not require special sample preparation, it has been considerd to be useful for biological applications, especially as an aid in medical diagnosis, and much research has been conducted. However, spectra derived from living organs contain a large amount of foreign information and are very difficult to analyze. Recent developments in AI and machine learning have provided one solution to this problem^15,21,22^. PCA is a statistical analysis method that aggregates data with many variables to create PCs to reduce the dimensionality of the data and facilitate comparison between data, and is a method frequently used in analysis of Raman spectral data as a type of unsupervised machine learning. In this study, we verified whether it is possible to detect amyloid deposition or discriminate between AL and ATTR based on the PCs obtained by PCA, and showed that the ROC curve had good diagnostic ability with an AUC of approximately 0.8. In the future, it is expected that more clinical data will be collected and supervised machine learning will be applied to enable even higher accuracy in diagnosis.

The problem still remains that diagnosis by AI and machine learning obscures the basis for the diagnosis, and explainable AI (XAI) would be one solution for this problem. Recently, XAI has been applied especially in the field of diagnostic imaging, and there is an increasing demand for XAI to show the basis of diagnosis^28,29^. However, XAI is not yet easy to introduce due to the complexity of the program, development costs, and other reasons. In such situation, it is also very important to add pathological or material science considerations to the differences in analysis of Raman spectra in order to obtain diagnostic certainty on a rational basis. We carefully examined the PCs obtained by PCA and the raw spectral data and found that the peak at 1680 cm^− 1^ and 1537 cm^− 1^ may be useful for detecting amyloid accumulation and discriminating between AL and ATTR, respectively. Furthermore, we tried to obtain theoretical support for the diagnosis based on these peaks by considering what molecular structure each peak is derived from.

We previously reported that the Raman peak assigned to the Amid-I bond near 1680 cm^− 1^ was useful for detecting amyloid deposition in the tissues of various organs, including the heart, in patients with AL amyloidosis^23^. Since a Raman spectroscopic analysis of immunoglobulins revealed a peak for the Amid-I bond^30,31^, we considered the peak for the Amid-I bond to be specific to AL amyloidosis. However, in the present study, the Raman peak at 1680 cm^− 1^ was still visible in amyloid-deposition regions, even in cardiac biopsy samples from ATTR amyloidosis patients. Therefore, we concluded that the 1680 cm^− 1^ peak was derived from the β-sheet structure of amyloid protein. Amyloid protein, which is deposited in tissues in amyloidosis, is a general term for a single protein that transforms into a β-sheet structure. The precursor proteins and peptides of amyloid proteins differ depending on the disease, with immunoglobulin light chains aggregating in AL and transthyretin aggregating in ATTR. Even though the precursor proteins differ, the β-sheet structure that is common to amyloid protein aggregates in a narrow region^24,25^, and the Raman peak at 1680 cm^− 1^ assigned to the β-sheet structure of the Amid-I bond is considered to be useful for detecting amyloid deposition areas with high sensitivity^32,33^.

In the present study, we analyzed the Raman spectra of amyloid deposition sites in detail and showed the possibility that differences in precursor proteins were reflected in Raman spectra. In a comparison of Raman spectra restricted to the amyloid deposition area, we found an apparent difference in Raman intensity between the AL and ATTR types of cardiac amyloidosis at the 1520–1540 cm^− 1^ wavenumber region. A detailed analysis by peak fitting also revealed a characteristic peak at 1537 cm^− 1^ in the AL type of cardiac amyloidosis, but not in the ATTR type. This difference in Raman spectra at 1537 cm^− 1^ may have been due to different amino acid sequences in the precursor proteins of AL and ATTR and, thus, we compared the amino acid sequences of the amyloid fibril-forming parts of immunoglobulin light chains and transthyretin. We found that the content of amino acids, which have a Raman peak near 1537 cm^− 1 26,27^, was nearly 10% higher in immunoglobulin light chains than in transthyretin. Since the intensity of the Raman spectra of each amino acid must also be considered and spectra may change due to peptide binding, it is still difficult to draw conclusions based on amino acid contents alone. Although studies comparing the Raman spectra of amino acids with those of peptides and proteins have been conducted since the development of Raman spectroscopy, differences in amino acid sequences and complex three-dimensional structures have made it difficult to predict changes in Raman spectra^34^. Attempts have been made to diagnose diseases using amino acids and peptides with characteristic spectra, such as aromatic amino acids and protonated phosphate groups, as indicators, but these have not been put to practical use^35,36^. It has been reported that by combining machine learning models, which have been remarkably developed in recent years, with classical force-field molecular dynamics, it has become possible to construct a model that predicts the Raman spectra of peptides from the Raman spectra of amino acids^37–39^. If more data are accumulated and such models are established in the future, it may be possible to predict variations in Raman spectra due to differences in precursor proteins in advance, and also to construct a model that discriminates disease types based on these predicted Raman spectra.

The prognosis of patients with systemic amyloidosis is negatively impacted by cardiac amyloidosis, which causes amyloid deposition in the heart. Even in non-fatal cases, cardiac amyloidosis is associated with repeated heart attacks and significantly impairs the quality of life of patients. Although the prognosis of cardiac amyloidosis has improved in recent years with the development of new therapies^2–6^, a prompt diagnosis is essential for the initiation of treatment as soon as possible in order to maximize therapeutic effects. Moreover, since treatment methods differ between AL and ATTR amyloidosis, it is important to rapidly diagnose not only the presence or absence of amyloid deposition, but also the disease type. Until now, research on the diagnosis of amyloidosis by Raman spectroscopy has mainly focused on the detection of amyloid protein. Kim et al. reported that Raman spectroscopy could not only identify the site of amyloid deposition in renal amyloidosis but also discriminate between amyloid A (AA) and AL types of amyloidosis^40^. This was one of the few studies to demonstrate the applicability of Raman spectroscopy to the classification of disease types in amyloidosis. To the best of our knowledge, the present study is the first to classify of the disease types of cardiac amyloidosis using Raman spectroscopy and demonstrate its potential. In the future, if it becomes possible to detect amyloid deposition based on the peak derived from the Amid-I bond at 1680 cm^− 1^ and further classify the disease type based on the peak around 1537 cm^− 1^ using a detailed spectral analysis of the same region, the diagnosis of cardiac amyloidosis may be expedited. Since Raman spectroscopy may analyze unstained specimens, a rapid diagnosis may be possible after a biopsy sample is taken, which will enable the early initiation of treatment.

There were several limitations that need to be addressed. The number of patients included was very small, 4 each for AL and ATTR, and it remains unclear whether a similar spectrum useful for diagnosis could be obtained when the patient number of cases increases. Another form of systemic amyloidosis that may be complicated by cardiac amyloidosis is AA amyloidosis due to chronic inflammation. AA amyloidosis was not involved in the present study. Furthermore, there are multiple forms of AL amyloidosis, such as the lambda and kappa types of immunoglobulin light chains, and ATTR amyloidosis, including the hereditary and wild types. Although it is necessary to diagnose these types in order to develop a precise treatment strategy for each patient, the number of cases in this study was small and we have not yet reached that level of classification. Further spectral analyses and the accumulation of more cases subjected to a Raman spectroscopic analysis are necessary in the future.

## Materials and Methods

### Collection of clinical biopsy samples

All clinical studies were approved by the Institutional Review Board of Tokushima University (approval number: 3507–3). All methods were performed in accordance with the guidelines and regulations of Tokushima University, which was created on the basis of Declaration of Helsinki. Written informed consent was obtained from patients for the collection of biopsy samples for diagnosis and the use of some specimens for research purposes. The background data of patients enrolled in the present study are summarized in Table 1. Cardiac biopsy samples were fixed in formalin and embedded in paraffin, and two or more serial sections (thickness of 3 μm) were prepared using a microtome. One of the serial sections was stained with Congo Red for polarized light microscopy and the adjacent sections were left unstained for Raman spectroscopy.

### Raman microscopy and image construction

Prior to Raman microscopy, Congo Red stained specimens were observed by polarized light microscopy (OPTIPHOT2-POL, Nikon Co.) to identify the area with apple green birefringence, which was presumed to be the amyloid deposition site. Based on the findings of polarized light microscopy, we identified the location for Raman spectroscopy as the region of interest.

Consecutive unstained specimens were observed by Raman spectroscopy (Raman11, Nanophoton Corporation, Osaka, Japan) as previously described^23^. The laser (central wavelength 532 nm, power 10–12 mW) was illuminated through an objective lens (LUCPLAN, x20, N.A. 0.45, Olympus Co., Tokyo, Japan) and sample observations were simultaneously performed through the same objective lens. Measurements were conducted in the x-y line scan mode. Briefly, the laser was expanded in the x-axis direction to illuminate the sample in a linear manner (exposure time 600 s), and the resulting spectral data were simultaneously acquired by 400 CCD array and scanned repeatedly in the y-axis direction 70–80 times. The diffraction grating and central wavenumber were 1200 lines/mm and 1850 cm^− 1^, respectively. The measurement range and wavenumber resolution were approximately 1350–2350 cm^− 1^ and 0.9 cm^− 1^, respectively. A final spatial resolution of 1 μm was achieved, and data including Raman spectra were acquired for each pixel. A Raman image was constructed based on spectral information of each pixel. The ratio of Raman scattering intensities in the wavenumber range of 1665–1680 cm^− 1^ to that in the wavenumber range of 1580–1600 cm^− 1^ was calculated, and the image was displayed with a pseudo-color gradient of black, magenta, cyan, and white from the lowest to highest ratio.

### PCA and creation of ROC curves

The Raman spectra of normal and amyloid-deposition areas were extracted, and numerical data were subjected to PCA. A two-dimensional scatter plot analysis was performed based on extracted PCs. ROC curves were constructed to evaluate sensitivity and specificity for predicting amyloid deposition or the discrimination of disease types based on PCA. PCA of the spectral data and a ROC curve analysis were performed using Python ver.3.10.9^41^. A more detailed spectral analysis within the wavenumber range of interest was conducted using multiple peak-fitting method by Igor Pro ver.9.0.5.1 (WaveMetrics)^23^.

### Amino acid sequence analysis

To investigate whether differences in the amino acid sequences of precursor proteins represent a difference in Raman spectra, we constructed the 3-dimensional structures of amyloid proteins of immunoglobulin light chains and transthyretin based on previous studies^24,25^, and colored several target amino acids in red using UCSF Chimera software^42^.

## Supplementary Material

This is a list of supplementary files associated with this preprint. Click to download.

SciRepRamanSupple20250601.docx

SciRepRamanFiguresSupple20250601.pdf

## Figures and Tables

**Table 1:** Patient data on AL and ATTR cardiac amyloidosis.

	AL (*n*=4)	ATTR (*n*=4)
Age	64.0 (49–74)	76.0 (65–88)
Male (Female)	1 (3)	3 (1)
Number of NYHA class III or IV	3	1
BNP [pg/mL]	1992.3 (65.5–5679.8)	231.4 (99.1–330.3)
LVDd [mm]	43.0 (33–54)	48.0 (46–50)
LVEF [%]	56.8 (48–66)	56.3 (45–62)
%FS [%]	28.0 (18–49)	27.5 (23–38)
IVST [mm]	12.1 (8.3–14.2)	12.7 (8.6–15.4)
LVPWT [mm]	11.9 (7.8–14.9)	12.5 (8.8–14.9)
LAD [mm]	45.0 (35–50)	46.8 (37–51)

NYHA class: New York Heart Association functional classification, BNP: brain natriuretic peptide, LVDd: diastolic left ventricular diameter, LVEF: left ventricular ejection fraction, %FS: fractional shortening, IVST: thickness of the interventricular septum, LVPWT: thickness of the left ventricular posterior wall, LAD: left atrial diameter.

## Data Availability

All data supporting the findings of this study are available within this draft manuscript.
